# Supplementary material for: The radiative feedback continuum from Snowball Earth to an ice-free hothouse
Source: Nat Commun. 2024 Aug 3;15:6582. doi: 10.1038/s41467-024-50406-w (PMC11297920; doi:10.1038/s41467-024-50406-w)
Supplement: Supplementary file 1 — Supplementary Information [file 41467_2024_50406_MOESM1_ESM.pdf]

Supplementary Information for "The radiative feedback continuum from Snowball Earth to an ice-free hothouse"

Ian Eisenman and Kyle C. Armour

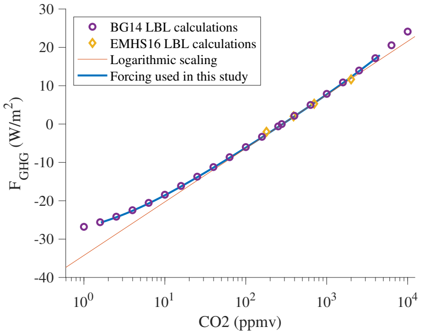

Figure S1: CO_2 radiative forcing. Circles indicate values from the Byrne and Goldblatt [1] supplemental data file "text03.txt", diamonds indicate values from the Etminan et al. [2] supplemental data table S1, the thin orange line indicates a logarithmic scaling of $F_{GHG} = F_{2\times} \log_2(C/C_0)$ with $F_{2\times} = 4.2$ W/m^2 and C the varying CO_2 concentration which is scaled by the PI value $C_0 = 284.7$ ppm, and the thick blue line indicates the CO_2 radiative forcing used in this study (see Methods section of main text). Here the data from Etminan et al. [2] includes their 4 runs with their default concentrations of CH_4 and N_20 and varied CO_2 concentrations, and both the data from Etminan et al. [2] and the data from Byrne and Goldblatt [1] are shifted vertically such that the forcing is zero at 284.7 ppm.

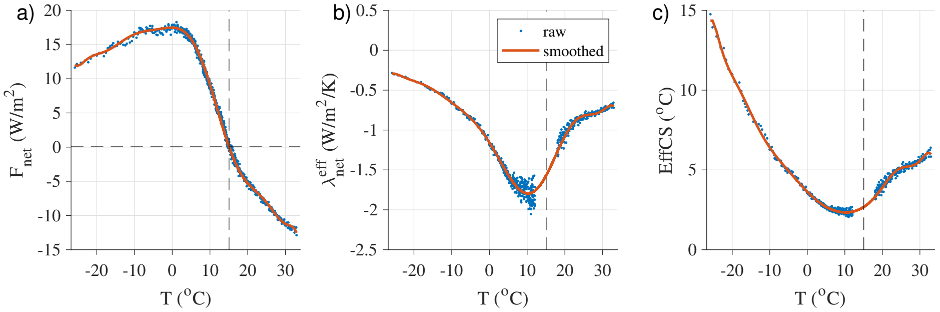

Figure S2: Smoothing of model output for $\lambda_{net}^{\text{eff}}$ calculation. (a) Net radiative response F_{net}. (b) Net effective feedback $\lambda_{net}^{\text{eff}}$. (c) Effective climate sensitivity EffCS. In each panel, the blue dots indicate the results with no smoothing of the raw annual-mean model output, and the red lines indicate the results after smoothing F_{net} with a 12th-order polynomial that is constrained to go through the PI reference climate value (intersection of black dashed lines in panel a). Because the numerator and denominator of Eq. 2 both asymptote to 0 at the PI climate, leading to large values of the ratio, the raw output is not plotted in panels b and c for T within 3K of the PI value.

S1 Idealized models

The interpretation of these results may be aided by considering idealized models that roughly mimic the CESM2 simulations. Here we consider first a single-layer model, and then a two-layer model, both of which are represented by simple ordinary differential equations.

Single-layer model. We begin with a single-layer model approximation to the terms in Eq. (1). We set $\Delta N = c\,\frac{dT}{dt}$, where t is time and $c = 15$ W yr/K/m^2 is the effective heat capacity describing the relationship between T and energy absorbed in the climate system with a value based on fitting the CESM2 results. We use a fourth-order polynomial approximation of the relationship between ΔF_{net} and T in Fig. 2c: $\Delta F_{net}(T) = p_1\,\Delta T + p_2\,\Delta T^2 + p_3\,\Delta T^3 + p_4\,\Delta T^4$, where we define ΔT as the departure from the PI value of T as in the calculation of $\lambda_{net}^{\mathrm{eff}}$; and similarly ΔF_{net} is the departure from the value in the PI climate, which we approximate to be zero such that $F_{net} = \Delta F_{net}$. The coefficients are $p_1 = -1.7$ W/m^2/K, $p_2 = 0.029$ W/m^2/K^2, $p_3 = 0.0042$ W/m^2/K^3, and $p_4 = 6.2\times10^{-5}$ W/m^2/K^4. This idealized representation of Eq. (1) takes the form of a nonlinear ordinary differential equation:

$$\frac{dT}{dt} = f(\Delta F_{GHG}, T) \equiv \frac{1}{c}\left[\Delta F_{GHG} + \Delta F_{net}(T)\right]. \tag{S1}$$

The associated feedback parameters $\lambda_{net}^{\mathrm{diff}}$ and $\lambda_{net}^{\mathrm{eff}}$ can be readily derived analytically in terms of the fit parameters in ΔF_{net}:

$$\lambda_{net}^{\mathrm{diff}} \equiv \frac{dF_{net}}{dT} = p_1 + 2\,p_2\,\Delta T + 3\,p_3\,\Delta T^2 + 4\,p_4\,\Delta T^3, \tag{S2}$$

$$\lambda_{net}^{\mathrm{eff}} \equiv \frac{\Delta F_{net}}{\Delta T} = p_1 + p_2\,\Delta T + p_3\,\Delta T^2 + p_4\,\Delta T^3. \tag{S3}$$

This system has steady-state solutions T^* that solve $0 = f(\Delta F_{GHG}, T^*)$, and the stability of these fixed points is dictated by $\frac{df}{dT} = \frac{1}{c}\frac{dF_{net}}{dT} = \frac{1}{c}\lambda_{net}^{\mathrm{diff}}$ evaluated at $T = T^*$. The idealized polynomial representation of $\lambda_{net}^{\mathrm{diff}}$ vs T in Eq. (S2) is shown in Fig. S3a.

Beginning from the fixed point with $\Delta F_{GHG} = 0$ (representing the PI), we increase and decrease the forcing as $\Delta F_{GHG} = \pm at$, with $a = \pm 0.055$ W/m^2/yr based on fitting the $\pm 1\%$ per year ramping of CO_2 in CESM2. The resulting time-evolving temperature is plotted versus the forcing in Fig. S3b (red line). Steady-state solutions are indicated in gray, with solid lines for stable solutions

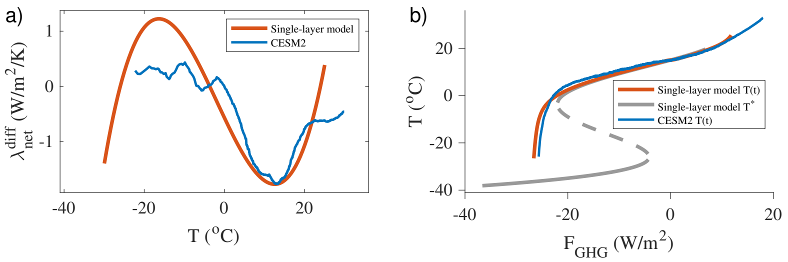

Figure S3: Single-layer idealized model result. (a) The polynomial representation of the feedback parameter $\lambda_{net}^{\rm diff}$ (Eq. (S2)) used in the idealized model (red), with the CESM2 results (as in Fig. 2d) included for comparison (blue). (b) The dependence of the temperature on the forcing. The time-evolving temperature simulated by the idealized model is shown in red. The steady-state solutions are shown in gray, with solid lines for stable solutions and a dashed line for the unstable solution. The CESM2 results are included for comparison (blue).

and a dashed line for the unstable solution. The CESM2 simulation results are included for comparison (blue line). Here the time-evolving temperature is computed from Eq. (S1) using numerical time stepping, and the steady-state solutions are computed using a polynomial root finder. It should be emphasized that this idealized model is presented as a tool to help explain the CESM2 results, rather than to add any quantitative information to the analysis; for example, the temperature associated with the cold stable state in Fig. S3b results from extrapolation outside the range of the CESM2 simulations and hence is sensitive to the details of the polynomial fit.

This helps to illustrate how the analysis used in this study does not depend on how equilibrated the climate system is with the evolving value of ΔF_{GHG}. Furthermore, the steady-state solutions of the ordinary differential equation can be readily found, indicating an unstable state at temperatures colder than the Snowball Earth bifurcation point, with a stable Snowball Earth state existing at even colder temperatures (beyond the range of climates simulated with CESM2). The time evolution of this simple system (Fig. S3b) helps illustrate how the positive values of $\lambda_{net}^{\rm diff}$ indicate times when the climate is transiently evolving across temperatures for which the only steady-state solution is unstable, rather than for example indicating an exponentially growing departure from an unstable climate state.

Two-layer model. We use the two-layer model of Held et al. [3], which takes

the form

$$c_s \frac{dT}{dt} = \Delta F_{GHG} + \lambda_0 \Delta T + \varepsilon \gamma (\Delta T_d - \Delta T) \tag{S4}$$

$$c_d \frac{dT_d}{dt} = \gamma (\Delta T - \Delta T_d). \tag{S5}$$

Here c_s is the heat capacity of the ocean surface layers that respond rapidly to the atmosphere, and we approximate this layer to be characterized by the surface temperature T, with ΔT the departure from the PI value of T as above; c_d and T_d are the heat capacity and temperature associated with the deeper ocean, with ΔT_d being the departure from the PI value of T_d; γ is a coefficient governing the heat-exchange between the two layers; λ_0 is a constant reference value of $\lambda_{net}^{\text{eff}}$; and ε is a factor to account for the deep ocean heat uptake efficacy associated with changes in the relationship between F_{net} and T due to the spatial pattern of surface temperature changes as the climate evolves toward equilibrium. Note that the extent to which $\varepsilon > 1$ represents the level of efficacy.

In this case the TOA net energy flux is equal to the change in heat content of the surface and deep layers, $\Delta N = c_s \frac{dT}{dt} + c_d \frac{dT_d}{dt} = c_s \frac{dT}{dt} + \gamma(\Delta T - \Delta T_d)$, and the deep ocean heat uptake efficacy influences the climate response as $\Delta F_{net} = \lambda_0 \Delta T - (\varepsilon - 1)\gamma(\Delta T - \Delta T_d)$. Note that here $F_{net} = \Delta F_{net}$ as for the single-layer model. With these relationships, Eqs. (S4)-(S5) are equivalent to Eq. (1). The resulting feedback parameters are

$$\lambda_{net}^{\text{diff}} \equiv \frac{dF_{net}}{dT} = \lambda_0 - (\varepsilon - 1)\gamma \left(1 - \frac{dT_d}{dT}\right) \tag{S6}$$

$$\lambda_{net}^{\text{eff}} \equiv \frac{\Delta F_{net}}{\Delta T} = \lambda_0 - (\varepsilon - 1)\gamma \left(1 - \frac{\Delta T_d}{\Delta T}\right). \tag{S7}$$

We use parameter values $\varepsilon = 1.28$, $\lambda_0 = -1.18$ W/m^2/K, $c_s = 8.2$ W yr/K/m^2, $c_d = 109$ W yr/K/m^2, and $\gamma = 0.67$ W/m^2/K, which are estimated from CMIP5 ensemble-mean simulation results [4]. As above, we begin from an equilibrated state and then increase and decrease the forcing as $\Delta F_{GHG} = \pm at$ with $a = \pm 0.055$ W/m^2/yr in order to mimic the $\pm 1\%$ per year ramping of CO_2 in CESM2. The time-evolving temperature is computed using numerical time stepping, and the term $\frac{dT_d}{dT}$ in Eq. (S6) is computed as the ratio of time derivatives, which we express in terms of the forcing and temperatures using Eq. (S4)-(S5). The resulting dependence of $\lambda_{net}^{\text{diff}}$ and $\lambda_{net}^{\text{eff}}$ on T is shown in Fig. S4 (red line). We also consider the impact of a large deep ocean heat uptake efficacy by using $\varepsilon = 2.5$ and adjusting the reference value of the feedback to $\lambda_0 = -0.5$ W/m^2/K,

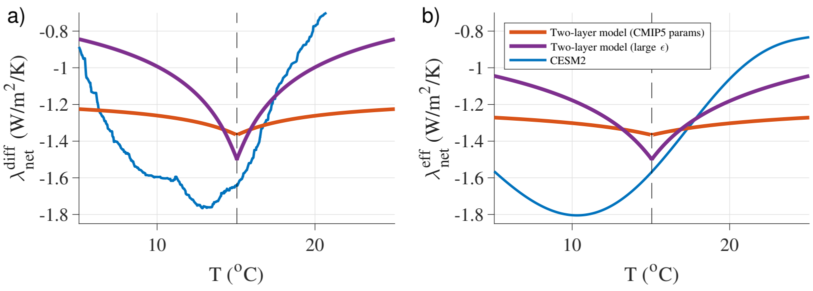

Figure S4: Two-layer idealized model results, showing the (a) differential and (b) effective net climate feedback. We include two parameter sets: moderate parameters estimated from the CMIP5 ensemble mean (red) and parameters adjusted to have a large deep ocean heat uptake efficacy (magenta). The CESM2 simulation results (as in Fig. 2d,e) are also included in blue.

which is plotted in magenta. The CESM2 simulation result is included for comparison (blue line).

S2 Radiative kernel analysis

The radiative kernel fields were computed by Pendergrass et al. [5] with the Parallel Offline Radiative Transfer model updated for compatibility with NCAR CAM5. The associated dataset includes monthly-mean radiative kernels associated with (1) surface temperature, (2) atmospheric temperature, (3) water vapor, and (4) surface albedo. The kernels, which vary as a function of space and time of year, represent the quantity $K_i \equiv \partial R/\partial v_i$, where R is the TOA net radiative response and v_i is the relevant component of the simulated climate. All kernels are set to zero above the tropopause, which is approximated as a linear function of the cosine of the latitude. The dataset also includes kernels computed using clear-sky radiative fields. We use the mean annual cycle averaged over years 480-499 of the PI simulation as the reference climate.

We define the annual-mean, zonal-mean, meridional-mean, and vertical-integration operations as

$$\langle\cdot\rangle_t \equiv \frac{1}{(1\text{ yr})}\int_0^{1\text{ yr}} \cdot\, dt,\ \ \langle\cdot\rangle_\theta \equiv \frac{1}{(360^\circ)}\int_0^{360^\circ} \cdot\, d\theta,$$
$$\langle\cdot\rangle_\phi \equiv \frac{1}{2}\int_{-90^\circ}^{90^\circ} \cdot\, w(\phi)\, d\phi,\ \ \{\cdot\}_p \equiv \int_{p_t}^{p_s} \cdot\, dp. \qquad \text{(S8)}$$

Here t is time, θ is longitude, ϕ is latitude, p is vertical pressure level, p_s is the surface pressure, p_t is the approximate tropopause pressure, and averages are performed on CESM2 model levels unless otherwise noted. Note that following Pendergrass et al. [5], we do not use the simulated varying p-field in the model, instead using a specified pressure field as a function of space and time of year based on a control simulation and the CAM hybrid grid. The gaussian weight $w(\phi) \approx \frac{\pi}{180^\circ} \cos\phi$ gives the area-weighting for each latitude; note that it departs slightly from a simple $\cos\phi$ scaling due to the details of the model grid. In what follows, a series of subscripts will indicate that series of averaging operations.

Note that although the CESM2 runs in the present study use CAM6, whereas the kernels are computed based on CAM5, both model versions have the same horizontal resolution. However, CAM6 has 32 levels and CAM5 has 30 levels, with the difference in vertical levels being confined exclusively to the stratosphere (the vertical levels are identical below the 88 hPa level). Since the kernel analysis is confined to the troposphere, the additional vertical resolution in the stratosphere in CAM6 does not require any interpolation of model fields.

The annual-mean global-mean radiative responses associated with each feedback (F_i), which are shown in Fig. S5, are computed by multiplying the monthly-mean simulation output during a given year with the radiative kernel and then averaging over time and space.

For the Planck feedback, this takes the form

$$\Delta F_P = \langle K_P(t,\theta,\phi)\, \Delta T_{s,2D}(t,\theta,\phi) \rangle_{t,\theta,\phi}, \tag{S9}$$

where K_P is the kernel and $T_{s,2D}$ is the surface temperature field.

For the lapse-rate feedback, it is

$$\Delta F_L = \left\langle \left\{ K_L(t,\theta,\phi,p)\, \Delta T_a'(t,\theta,\phi,p) \right\}_p \right\rangle_{t,\theta,\phi}, \tag{S10}$$

where K_L is the kernel and $\Delta T_a'$ is the departure of the 3D temperature change from the surface temperature change.

We use the "logarithmic" water-vapor kernel K_w in the Pendergrass et al. [5] dataset, for which the radiative response takes the form

$$\Delta F_w = \left\langle \left\{ K_w(t,\theta,\phi,p)\, \Delta Q(t,\theta,\phi,p) \,/\, \left[\frac{\Delta Q}{\Delta T_a}\right]_h \right\}_p \right\rangle_{t,\theta,\phi}, \tag{S11}$$

where the term $\left[\frac{\Delta Q}{\Delta T_a}\right]_h$ describes the change in specific humidity under constant relative humidity and is a function of the 3D temperature field T_a, and the kernel K_w gives the change in TOA radiation per change in atmospheric temperature

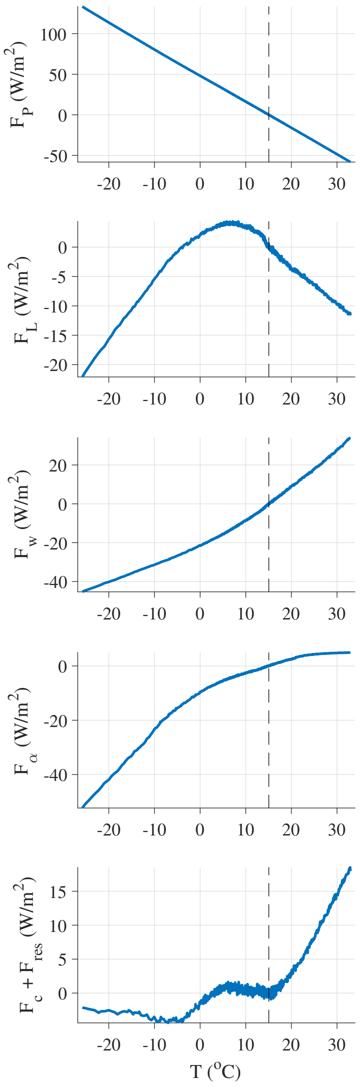

Figure S5: Radiative response associated with each feedback, computed using the radiative kernels.

that would occur if the specific humidity Q increased with the relative humidity h remaining as it is in the reference climate. Note that this representation does account for changes in relative humidity: there is essentially a normalization factor associated with constant relative humidity that is multiplied into K_w and divided out of the simulated field, such that it cancels in Eq. (S11).

For the albedo feedback, the radiative response is

$$\Delta F_\alpha = \langle\, K_\alpha(t,\theta,\phi)\,\Delta\alpha(t,\theta,\phi)\,\rangle_{t,\theta,\phi}\,, \tag{S12}$$

where K_α is the kernel and $\Delta\alpha$ is the change in the surface albedo, which is computed as $\alpha = S_{up}/S_{down}$ with S_{up} and S_{down} the upward and downward shortwave radiation at the surface.

S3 Approximating with averaged kernels

In the "Physical interpretation of feedback changes" section of main text, we interpret the results with the aid of a simplified analysis that uses annual-mean zonal-mean radiative kernels and simulated fields. We define the annual and zonal average of the kernels as

$$\begin{aligned} &\tilde{K}_P(\phi) \equiv \langle K_P(t,\theta,\phi)\rangle_{t,\theta}\,,\ \ \tilde{K}_L(\phi,p) \equiv \langle K_L(t,\theta,\phi,p)\rangle_{t,\theta}\,,\\ &\tilde{K}_w(\phi,p) \equiv \langle K_w(t,\theta,\phi,p)\rangle_{t,\theta}\,,\ \ \tilde{K}_\alpha(\phi) \equiv \langle K_\alpha(t,\theta,\phi)\rangle_{t,\theta}\,, \end{aligned} \tag{S13}$$

and we similarly define the annual and zonal average of the relevant simulated climate fields as

$$\tilde{T}_s(\phi) \equiv \langle T_{s,2D}(t,\theta,\phi)\rangle_{t,\theta}\,,\ \tilde{T}_a(\phi,p) \equiv \langle T_a(t,\theta,\phi,p)\rangle_{t,\theta}\,,\ \tilde{\alpha}(\phi) \equiv \frac{\langle S_{up}(t,\theta,\phi)\rangle_{\phi,p}}{\langle S_{down}(t,\theta,\phi)\rangle_{\phi,p}}. \tag{S14}$$

Note that for the annual-mean zonal-mean albedo field $\tilde{\alpha}$, this uses the ratio of the means rather than the mean of the ratio, which is important for the approximate match between Fig. 3 and Fig. S6.

For the water-vapor feedback, we further approximate that the relative humidity remains as in the reference climate. Under this approximation, the terms in Eq. (S11) involving humidity simplify to $\Delta Q \,/\, \left[\frac{\Delta Q}{\Delta T_a}\right]_h = \Delta T_a$.

The resulting approximate radiative responses, which we indicate as $\tilde{F}_i$, are

$$\Delta\tilde{F}_P = \left\langle \tilde{K}_P(\phi)\,\Delta\tilde{T}_s(\phi)\right\rangle_\phi,\;\; \Delta\tilde{F}_L = \left\langle \left\{\tilde{K}_L(\phi,p)\,\Delta\tilde{T}_a'(\phi,p)\right\}_p\right\rangle_\phi,$$
$$\Delta\tilde{F}_w = \left\langle \left\{\tilde{K}_w(\phi,p)\,\Delta\tilde{T}_a(\phi,p)\right\}_p\right\rangle_\phi,\;\; \Delta\tilde{F}_\alpha(\phi) = \left\langle \tilde{K}_\alpha(\phi)\,\Delta\tilde{\alpha}(\phi)\right\rangle_\phi, \quad \text{(S15)}$$

where $\Delta\tilde{T}_a'(\phi,p) \equiv \Delta\tilde{T}_a(\phi,p) - \Delta\tilde{T}_s(\phi)$ is the departure of the atmospheric temperature change from the surface temperature change.

The feedback parameters $\tilde{\lambda}_i$ are computed from these approximate radiative responses as above in Eq. (6) of the main text. The residual term, $\tilde{\lambda}_c + \tilde{\lambda}_{res}$, is computed as above using ΔN and ΔF_{GHG}, with the radiative responses F_i replaced with the annual-mean zonal-mean analysis values $\tilde{F}_i$. This leads to feedback parameter values $\tilde{\lambda}_i$ that match fairly closely with the feedback parameters λ_i that were computed using the full 4D structure of the simulated climate and kernel fields (Fig. S6).

Planck feedback. The outgoing radiation can be written according to the Stefan-Boltzmann law as $\varepsilon\sigma\tilde{T}_s^4$, where ε is the effective emissivity associated with the atmosphere making the surface less efficient at emitting radiation to space. The kernel K_P describes the change in incoming radiation per change in surface temperature, and hence the annual-mean zonal-mean kernel can be written as

$$\tilde{K}_P(\phi) = -4\,\varepsilon(\phi)\,\sigma\left[\tilde{T}_{s,PI}(\phi)\right]^3, \quad \text{(S16)}$$

where $\tilde{T}_{s,PI}$ indicates the annual and zonal average of the surface temperature field $\tilde{T}_s$ in the reference climate. The effective emissivity ε varies in space due to factors including cloudiness, but the kernel can be fairly well approximated using a uniform value of $\varepsilon = 0.61$, which is based on matching the global-mean values of $\tilde{K}_P(\phi)$ and $\left[\tilde{T}_{s,PI}\right]^3$. Hence the kernel $\tilde{K}_P$ has a more-negative value in locations with a warmer surface temperature in the reference climate.

The differential Planck feedback parameter can be written as

$$\tilde{\lambda}_P^{\text{diff}} = \frac{\Delta\tilde{F}_P}{\Delta T} \approx \left\langle \tilde{K}_P(\phi)\,\frac{\Delta\tilde{T}_s(\phi)}{\Delta T}\right\rangle_\phi = \left\langle \tilde{K}_P(\phi)\right\rangle_\phi + \left\langle \tilde{K}_P(\phi)\,\frac{\Delta\tilde{T}_s(\phi)-\Delta T}{\Delta T}\right\rangle_\phi, \quad \text{(S17)}$$

which shows that the feedback is equal to the global-mean value of the kernel, plus a correction associated with locations where the temperature change departs from the global-mean temperature change. The evolution of the annual-mean zonal-mean surface temperature $\tilde{T}_s(\phi)$ is plotted in Fig. S7. The temperature departure

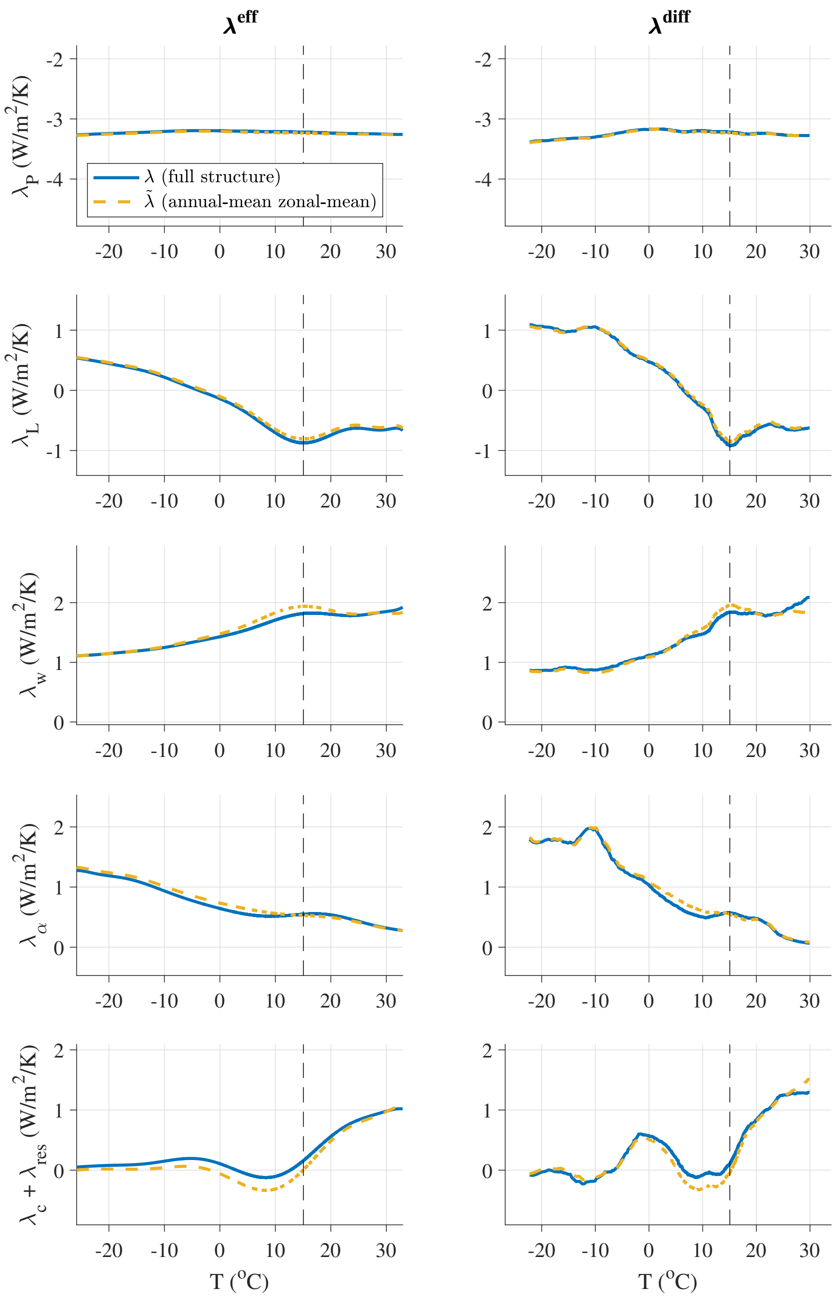

Figure S6: As in Fig. 3, but also including the results computed using annual-mean zonal-mean kernels and simulated climate fields (orange dashed lines).

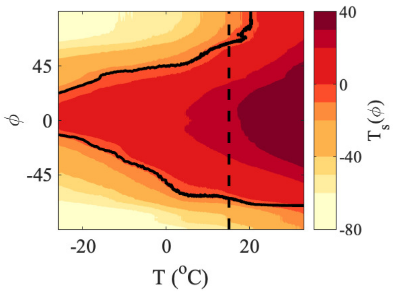

Figure S7: Meridional structure of simulated surface temperature changes. The black solid line indicates the 50% contour of the ice cover, and the black vertical dashed line indicates the PI climate.

term in Eq. (S17), $\frac{\Delta\tilde{T}_s(\phi)-\Delta T}{\Delta T}$, is plotted in Fig. 4f. This term is computed from $\tilde{T}_s(\phi)$ using the same TLS regression procedure as in the computation of the differential feedback parameters described in the Methods section of the main text. Note that the approximately equal sign in Eq. (S17) indicates that the TLS regression operation is being approximated as linear (OLS regression is linear whereas TLS regression is not).

Lapse-rate feedback. The lapse-rate feedback parameter can be written as

$$\tilde{\lambda}_L^{\mathrm{diff}} = \frac{\Delta\tilde{F}_L}{\Delta T} \approx \left\langle \left\{ \tilde{K}_L(\phi,p)\,\frac{\Delta\tilde{T}_a'(\phi,p)}{\Delta T} \right\}_p \right\rangle_\phi . \qquad \text{(S18)}$$

The quantity inside the meridional averaging operation is plotted in Fig. 4b. As in Eq. (S17), the ratio is computed using TLS regression, and the approximately equal sign indicates that this operation is being approximated as linear.

We repeat the analysis neglecting horizontal variations in the temperature profile (and kernel), in which case the parameter is approximated as

$$\tilde{\lambda}_L^{\mathrm{diff}} \approx \left\{ \left\langle \tilde{K}_L(\phi,p) \right\rangle_\phi \left\langle \frac{\Delta\tilde{T}_a'(\phi,p)}{\Delta T} \right\rangle_\phi \right\}_p . \qquad \text{(S19)}$$

Since the meridional average is performed before the vertical integration for the calculation in Eq. (S19), we carry out this meridional average on pressure levels rather than on model levels. The result is plotted in Fig. 4c (red line), which shows that changes in the globally-averaged temperature profile dominate the variations in the lapse-rate feedback parameter.

Albedo feedback. The radiative response associated with the albedo feedback can be written as

$$\tilde{F}_\alpha(\phi) = \underbrace{\left\langle \tilde{K}_\alpha(\phi) \right\rangle_\phi \langle \tilde{\alpha}(\phi) \rangle_\phi}_{\text{constant } \tilde{K}_\alpha} + \underbrace{\left\langle \left(\tilde{K}_\alpha(\phi) - \left\langle \tilde{K}_\alpha(\phi) \right\rangle_\phi \right) \tilde{\alpha}(\phi) \right\rangle_\phi}_{\text{effect of } \tilde{K}_\alpha \text{ variations}}. \quad \text{(S20)}$$

The first term on the right-hand side describes the influence of changes in the global-mean albedo alone, and the second term describes the effect of higher levels of incident solar radiation in low latitudes (as well as other factors that cause spatial variations in the kernel). The first term is scaled by the global-mean value of the kernel, $\left\langle \tilde{K}_\alpha(\phi) \right\rangle_\phi = -140$ W/m^2, whose magnitude is about 40% of the global-mean insolation, 340 W/m^2. If we neglect spatial variations in the kernel, the albedo feedback parameter can be approximated using the first term in Eq. (S20) alone as

$$\tilde{\lambda}_\alpha^{\text{diff}} \approx \left\langle \tilde{K}_\alpha(\phi) \right\rangle_\phi \frac{\Delta \langle \tilde{\alpha}(\phi) \rangle_\phi}{\Delta T}. \quad \text{(S21)}$$

This can be further simplified by using the relationship $\Delta \langle \tilde{\alpha}(\phi) \rangle_\phi \approx \delta_\alpha \Delta A_{ice}$. Here A_{ice} is the global ice area that is plotted in Figs. 1c and 4d, which includes sea ice, snow cover on land, and glacial ice, and is measured as a fraction of the globe; and $\delta_\alpha = 0.72$ is the surface albedo jump, which is determined here by regression between ice area A_{ice} and global-mean albedo $\langle \tilde{\alpha}(\phi) \rangle_\phi$. Inserting this into Eq. (S21) leads to

$$\tilde{\lambda}_\alpha^{\text{diff}} \approx \left\langle \tilde{K}_\alpha(\phi) \right\rangle_\phi \delta_\alpha \frac{\Delta A_{ice}}{\Delta T}. \quad \text{(S22)}$$

In this representation, the albedo feedback parameter is approximated as the sensitivity of the total ice area to global mean temperature, which is the slope $\frac{\Delta A_{ice}}{\Delta T}$ in Fig. 4d, scaled by a constant value. Fig. 4e shows that this approximation captures much of the variation in the albedo feedback parameter.

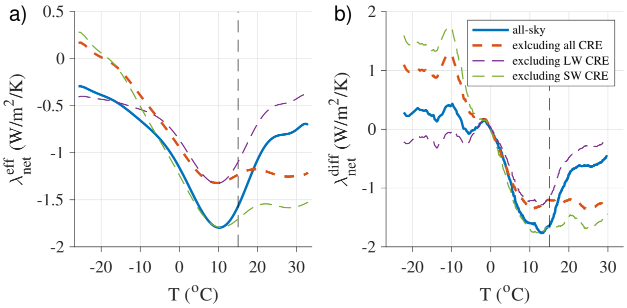

Figure S8: As in Fig. 2d,e, but including results computed using clear-sky fields. The net feedback parameter shown in Fig. 2d,e (shown here as blue curves) is calculated using Eq. (2) in the main text, which can be written as $\lambda_{net} \equiv \Delta F_{net} / \Delta T = \Delta(\texttt{FSNT} - \texttt{FLNT} - F_{GHG}) / \Delta T$, where `FSNT` and `FLNT` are the top-of-model longwave (LW) and shortwave (SW) fluxes reported by the atmospheric model. We exclude cloud radiative effects (CRE) by replacing `FSNT` and `FLNT` with clear-sky fields reported by the model (`FSNTC` and `FLNTC`), which is indicated by the red dashed lines. Next we exclude only LW or SW CRE by replacing only `FLNT` (magenta) or only `FSNT` (green) with clear-sky fields.

References

[1] Byrne, B. & Goldblatt, C. Radiative forcing at high concentrations of well-mixed greenhouse gases. *Geophys. Res. Lett.* **41**, 152–160 (2014).

[2] Etminan, M., Myhre, G., Highwood, E. J. & Shine, K. P. Radiative forcing of carbon dioxide, methane, and nitrous oxide: A significant revision of the methane radiative forcing. *Geophys. Res. Lett.* **43**, 12614–12623 (2016).

[3] Held, I. M. *et al.* Probing the fast and slow components of global warming by returning abruptly to preindustrial forcing. *J. Climate* **23**, 2418–2427 (2010).

[4] Geoffroy, O. *et al.* Transient climate response in a two-layer energy-balance model. Part II: Representation of the efficacy of deep-ocean heat uptake and validation for CMIP5 AOGCMs. *J. Climate* **26**, 1859–1876 (2013).

[5] Pendergrass, A. G., Conley, A. & Vitt, F. M. Surface and top-of-atmosphere radiative feedback kernels for CESM-CAM5. *Earth System Sci. Data* **10**, 317–324 (2018).
